# Dietary inflammatory index and elevated serum C‐reactive protein: A systematic review and meta‐analysis

**DOI:** 10.1002/fsn3.3553

**Published:** 2023-07-06

**Authors:** Salman Mohammadi, Mahboobe Hosseinikia, Ali Ghaffarian‐Bahraman, Cain C. T. Clark, Ian G Davies, Esmaeil Yousefi Rad, Somayeh Saboori

**Affiliations:** ^1^ Nutritional Health Research Center Lorestan University of Medical Sciences Khorramabad Iran; ^2^ Department of Nutrition and Food Sciences Yasuj University of Medical Sciences Yasuj Iran; ^3^ Occupational Environment Research Center Rafsanjan University of Medical Sciences Rafsanjan Iran; ^4^ Centre for Intelligent Healthcare Coventry University Coventry UK; ^5^ Research Institute of Sport and Exercise Science Liverpool John Moores University Liverpool UK; ^6^ Oxford Brookes Centre for Nutrition and Health (OxBCNH) Department of Sport, Health Sciences and Social Work, Faculty of Health and Life Sciences Oxford Brookes University Oxford UK

**Keywords:** CRP, dietary indices, dietary inflammation index, DII

## Abstract

Diet can affect the inflammatory state of the body. Accordingly, the dietary inflammatory index (DII) has been developed to quantify the inflammatory properties of food items. This study sought to investigate the association between dietary inflammation index (DII) and the odds ratio of elevated CRP (E‐CRP) through a systematic review and meta‐analysis study. The International electronic databases of PubMed, Web of Science (ISI), and Scopus were searched until May 2023 to find related articles. From 719 studies found in the initial search, 14 studies, with a total sample size of 59,941 individuals, were included in the meta‐analysis. The calculated pooled odds ratio (OR) of E‐CRP in the highest DII category was 1.39 (95% CI: 1.06, 1.14, test for heterogeneity: *p* = .63, and *I* ^2^ = .0%) in comparison with the lowest DII category. Also, the results of this study showed that each unit increase in DII as a continuous variable generally elicited a 10% increase in the odds of E‐CRP (OR 1.10, 95% CI 1.06, 1.14, test for heterogeneity: *p* = .63, and *I* ^2^ = .0%). Subgroup meta‐analyses showed that there is a higher E‐CRP odds ratio for the articles that reported energy‐adjusted DII (E‐DII) instead of DII, the studies that measured CRP instead of hs‐CRP, and the studies that used 24‐h recall instead of FFQ as the instrument of dietary intake data collection. Individuals with a higher DII were estimated to have higher chances of developing elevated serum CRP. This value was influenced by factors such as the participants' nationality, instruments of data collection, methods used to measure inflammatory biomarkers, study design, and data adjustments. However, future well‐designed studies can help provide a more comprehensive understanding of the inflammatory properties of diet and inflammatory serum biomarkers.

## Graphical abstract

This systematic review and meta‐analysis examined the relationship between the dietary Inflammatory Index (DII) and the odds of elevated CRP (E‐CRP). The findings suggest that higher DII scores are associated with increased odds of E‐CRP, indicating a potential link between dietary inflammation and elevated serum CRP levels. Further well‐designed studies are needed to explore the intricate relationship between diet, inflammatory biomarkers, and their impact on health outcomes.

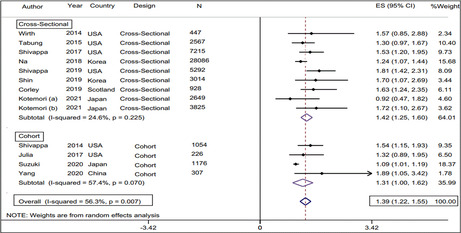

## 1 INTRODUCTION

Inflammation is a controlled physiological response that the body exhibits to defend against tissue damage or infection. Moreover, the continuous release of inflammatory mediators in the serum can lead to tissue damage (Medzhitov, 2008). Chronic inflammation plays a role in the pathogenesis of different disorders, including diabetes mellitus, obesity, depression, cancer, and cardiovascular diseases (Guo et al., 2013; Haghani et al., 2022; Kiecolt‐Glaser et al., 2015; Sarwar et al., 2009; Smidowicz & Regula, 2015). There are various biomarkers to evaluate the inflammatory state of the body, the most important of which are serum C‐reactive protein (CRP) and cytokines, including interleukins, interferon, and tumor necrosis factor (Calder et al., 2013).

Empirical studies have shown that serum levels of inflammatory biomarkers strongly correlate to lifestyle variables, such as diet, smoking, and physical activity. Dietary components are among the most essential key factors in regulating the inflammatory state of the body, and, according to previous investigations, a western diet rich in red meat, whole‐fat dairy, refined grains, and refined carbohydrates may be associated with higher levels of serum CRP (Ley et al., 2014; Lopez‐Garcia et al., 2004). In contrast, consumption of a Mediterranean diet, rich in whole grains, fish, fruit, and olive oil, has been associated with lower levels of inflammatory biomarkers (Esposito et al., 2004; Serrano–Martinez et al., 2005; Wannamethee et al., 2006).

The dietary inflammatory index (DII) was developed based on the association between 45 dietary components and food items (monounsaturated fatty acids, polyunsaturated fatty acids, n‐3 fatty acids, n‐6 fatty acids, fiber, alcohol, vitamins A, D, E, C, and B_6_, β‐carotene, thiamine, riboflavin, niacin, folic acid, magnesium, selenium, zinc, flavan‐3‐ol, flavones, flavonols, flavanones, anthocyanidins, isoflavones, pepper, thyme/oregano, rosemary, turmeric, saffron, ginger, energy, eugenol, caffeine, garlic, onion, and green/black tea, carbohydrates, protein, total fat, saturated fatty acids, trans fat, cholesterol, iron, and vitamin B_12_) with six inflammatory biomarkers including IL‐1β, IL‐4, IL‐6, IL‐10, TNF‐α, and CRP, through a comprehensive literature review of the studies published from 1950 to 2010. In fact, this index is the sum of the positive and negative scores attributed to each of the aforementioned dietary components indicating their pro‐inflammatory or anti‐inflammatory potentials, respectively. A higher DII score represents a more inflammatory diet (Shivappa, Steck, Hurley, Hussey, & Hébert, 2014b).

So far, several studies have investigated the relationship between DII and the inflammatory biomarker CRP in different populations. However, there are contradictions in the findings of these studies. To the best of our knowledge, no previous systematic review and meta‐analysis have investigated the association between DII and the odds ratio of elevated serum C‐reactive protein (E‐CRP). Therefore, in the present study, we aimed to assess the odds ratio of E‐CRP in relation to the dietary inflammation index in different population subgroups created based on gender, nationality, study design and methods of measuring laboratory biomarkers, data collection, and variable adjustments.

## 2 MATERIALS AND METHODS

### 2.1 Search strategy and study selection

The current study was performed based on MOOSE Guidelines for Meta‐Analyses and Systematic Reviews of Observational Studies (Brooke et al., 2021). A comprehensive literature search was performed by two independent qualified investigators (MH and SS) under the supervision of third reviewer (EY) in the online databases of PubMed, Scopus, and Web of Sciences, until March 2022 and then updated on May 2023. The following keywords were used in the search strategy to be found in titles, abstracts, and keywords of the studies: (“Dietary inflammatory index” OR “inflammatory diet” OR “anti‐inflammatory diet” OR “dietary score” OR DII OR “pro‐inflammatory diet” OR “inflammatory potential of diet”) AND (“C‐reactive protein” OR “high‐sensitivity CRP” OR “hs‐CRP” OR CRP) (Table S1). No restriction was imposed on time of publication or language. The reference list of the relevant articles was also reviewed to avoid missing any potentially relevant publications. Having removed duplicate citations, all remaining studies in the initial search were screened by their titles and abstracts, and eligible studies underwent full‐text review by two reviewers.

### 2.2 Inclusion and exclusion criteria

We included studies if they met the following criteria: (1) observational studies with cohort, case–control, or cross‐sectional designs; (2) Investigated the relationship between DII and CRP as the outcome variable in a wide range of clinical settings; (3) performed on a population of adults (≥18 years); (4) reported odds ratios [ORs], risk ratios [RRs], or hazard ratios [HRs] along with their 95% confidence intervals [CIs] for the association between DII and CRP. If findings from one study were published in more than one article and/or database, we selected the most credible version. We excluded letters, comments, short communications, review articles, and ecological and animal studies.

### 2.3 Data extraction

Required data from each eligible study were extracted by two independent investigators (MH and SS), and any disagreements were reconciled by discussion or referral to a third reviewer (EY). Any reported ORs or HRs or RRs and corresponding 95% CIs for the association between DII and CRP were extracted from each study. In addition to effect sizes (ESs), the following information was extracted: first author's name, year of publication, country of origin, demographic characteristics of participants (age range and gender), number of participants and cases, duration of follow‐up for prospective studies, methods used for exposure and outcome assessment, and confounding variables adjusted in the statistical analysis. All extracted data were included in a standardized Microsoft Excel.

### 2.4 Data synthesis and statistical analysis

For comparison of the highest versus lowest categories of DII, ORs and RRs (along with 95% CIs) were used to calculate weighted ORs and RRs. To estimate the pooled effect size, random effects model was used for analyses. Existence of heterogeneity was checked by Cochran's *Q*‐test and *I* ^2^ test. *I* ^2^ values of >50% were considered significant between‐study heterogeneity. Publication bias was examined using funnel plot visual inspection and Egger's regression asymmetry tests. A trim‐and‐fill method was used to detect the effect of probable missing studies on the overall effect. All statistical analyses were performed using Stata version 14.0 (Stata Corporation, College Station). All *p*‐values were two‐sided, and *p* < .05 was considered statistically significant.

### 2.5 Quality assessment

As illustrated in Table S2, the quality of included studies in the current meta‐analysis was assessed by two independent authors using the Newcastle Ottawa Scale (NOS), designed for observational studies (Peterson et al., 2011; Stang, 2010). According to this scale, a maximum of 9 points could be awarded to each study according to the following parameters: 4 points for the selection of participants, 2 points for comparability, and 3 points for the assessment of outcomes. A study with score from 7 to 9 has high quality, 4–6 has high risk, and 0–3 has very high risk of bias.

## 3 RESULTS

**FIGURE 1:**
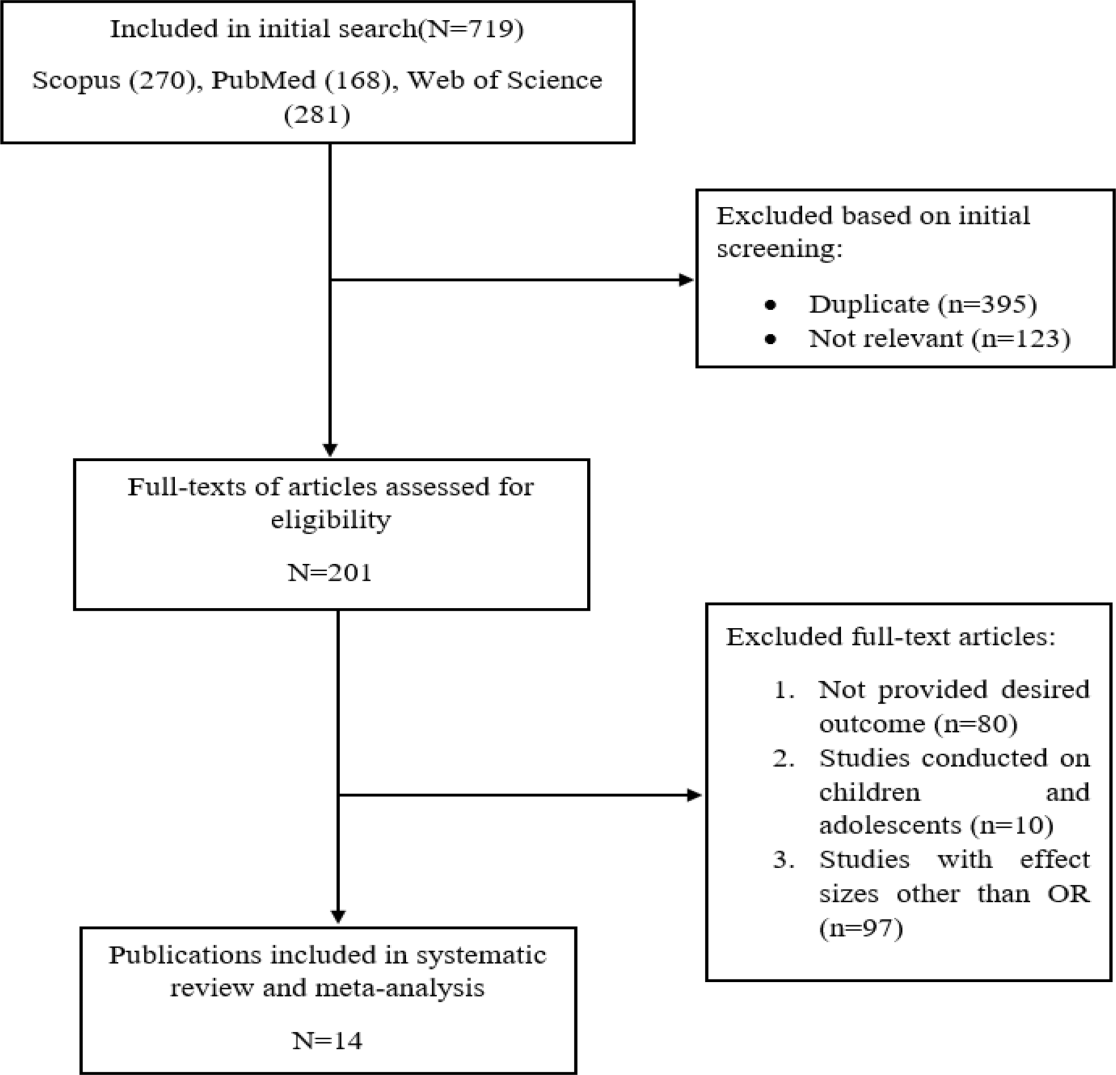
Flowchart of the study selection for inclusion in the systematic.

During the initial step of our meta‐analysis search, a total of 719 publications were identified. Among these, 395 articles were removed due to duplication, and an additional 123 studies were excluded based on their titles and abstracts as they were deemed irrelevant. Following careful screening, 201 publications were selected for full‐text eligibility assessment. After a thorough evaluation, we included 14 clinical trials in this systematic review and meta‐analysis, meeting our predefined inclusion criteria (Figure 1).

Fourteen included studies in this systematic review encompassed a diverse range of participants, with the total number of individuals varying from 226 to 28,086 across all studies. Consequently, a total of 59,941 participants were enrolled in these studies. Among the included articles, 11 encompassed both genders, while three studies specifically focused on women.

These studies originated from a limited number of countries, including the United States (Julia et al., 2017; Shin et al., 2017; Shivappa et al., 2019; Shivappa, Steck, Hurley, Hussey, Ma, et al., 2014a; Tabung et al., 2015; Wirth et al., 2014), Japan (Kotemori et al., 2021; Suzuki et al., 2020), Korea (Na et al., 2018; Shin et al., 2019), China (Yang et al., 2020), Belgium (Shivappa et al., 2015), and Scotland (Corley et al., 2019).

Assessment of the dietary inflammatory index was conducted using food frequency questionnaires (FFQs) in eight papers, providing a comprehensive overview of participants' long‐term dietary habits. Additionally, 24‐h dietary recall (24HR) was employed in five articles, offering a snapshot of participants' dietary intake within a specific 24‐h period. Further details regarding the main characteristics of the included studies can be found in Table 1.

**TABLE 1:** Characteristics of the included studies of dietary inflammatory index and C‐reactive protein.

First author (year)	Country	Study design	Exposure type	Dietary assessment/number of food parameter for DII calculation	Gender of participants	Sample size	Population	Comparison	Adjustments/matching	NOS stars
Corley et al. (2019)	Scotland	Cross‐sectional	E‐DII	FFQ‐26	Both	928	Older Adults	T3 versus T1 DII‐continuous	Energy intake, age, sex, BMI, smoking, physical activity, hypercholesterolemia	9
Julia et al. (2017)	USA	Cohort	DII/E‐DII	FFQ‐36	Both	226	Healthy people	T3 versus T1	Energy intake, sex, age, education level, smoking, physical activity, BMI, number of dietary records available	8
Kotemori et al. (2021)	Japan	Cross‐sectional	E‐DII	FFQ‐30	Both	6474	Healthy people	Q4 versus Q1	Age, BMI, physical activity, smoking	10
Na et al. (2018)	Korea	Cross‐sectional	DII	FFQ‐10	Both	28,086	Healthy people	Q4 versus Q1	Age, sex, BMI, Smoking, education, BP, energy intake	8
Shin et al. (2019)	Korea	Cross‐sectional	DII	24HR‐23	Both	3014	Healthy people	Q5 versus Q1 DII‐continuous	Age, sex, education, marital status, smoking, alcohol consumption, BMI, HDL, physical activity	10
Shin et al. (2017)	USA	Cross‐sectional	DII	24HR‐27	Female	631	Pregnant women	DII‐continuous	Age, family income, month in pregnancy, race, ethnicity, education, smoking	7
Shivappa et al. (2017)	USA	Cross‐sectional	DII	24HR‐27	Both	7215	Healthy people	Q4 versus Q1 DII‐continuous	Age, sex, ethnicity, BMI, education, smoking, poverty index, and physical activity	10
Shivappa et al. (2019)	USA	Cross‐sectional	E‐DII	24HR‐27	Both	5292	Healthy people	Q4 versus Q1	Age, sex, ethnicity, BMI, poverty index	10
Shivappa (2014a, 2014b)	USA	Cohort	DII	24HR‐44 7DDR‐28	Both	495 559	Healthy people	T3 versus T1 DII‐continuous	MET, gender, light season, race, marital status, serum total cholesterol, employment status, anti‐inflammatory medication use, alcohol status, and herbal supplement use.	7
Shivappa et al. (2015)	Belgium	Cross‐sectional	DII	FFQ‐17	Both	2524	Healthy people	DII‐continuous	Energy, age, sex, BMI, education, use of NSAID, BP, use of OCP, antihypertensive therapy, lipid‐lowering drugs, and physical activity.	10
Suzuki et al. (2020)	Japan	Cohort	DII	FFQ‐26	Both	1176	Healthy people	Q4 versus Q1 DII‐continuous	Sex, age, smoking, drinking habits, history of hypertension, energy intake, BMI.	8
Tabung et al. (2015)	USA	Cross‐sectional	DII	FFQ‐32	Female	2567	Healthy people	Q5 versus Q1	Age, BMI, race, educational level, smoking, physical activity, inflammation‐related comorbidity, regular use of antidepressants, Statins, and NSAIDs	9
Wirth et al. (2014)	USA	Cross‐sectional	DII	FFQ‐27	Both	447	Healthy people	Q4 versus Q1	Age, education	6
Yang et al. (2020)	China	Cohort	DII	24HR‐45	Female	307	Pregnant women (16–20 weeks of pregnancy)	T3 versus T1	–	6

Abbreviations: 24HR, 24‐h dietary recall; BMI, body mass index; BP, blood pressure, DII, dietary inflammatory index; E‐DII, Energy‐adjusted dietary inflammatory index; FFQ, food frequency questionnaire; HDL, high‐density lipoprotein; MET, metabolic equivalent of task; NSAID, nonsteroidal anti‐inflammatory drugs; OCP, oral contraceptives; SBP, Systolic blood pressure.

### 3.1 Meta‐analysis

**FIGURE 2:**
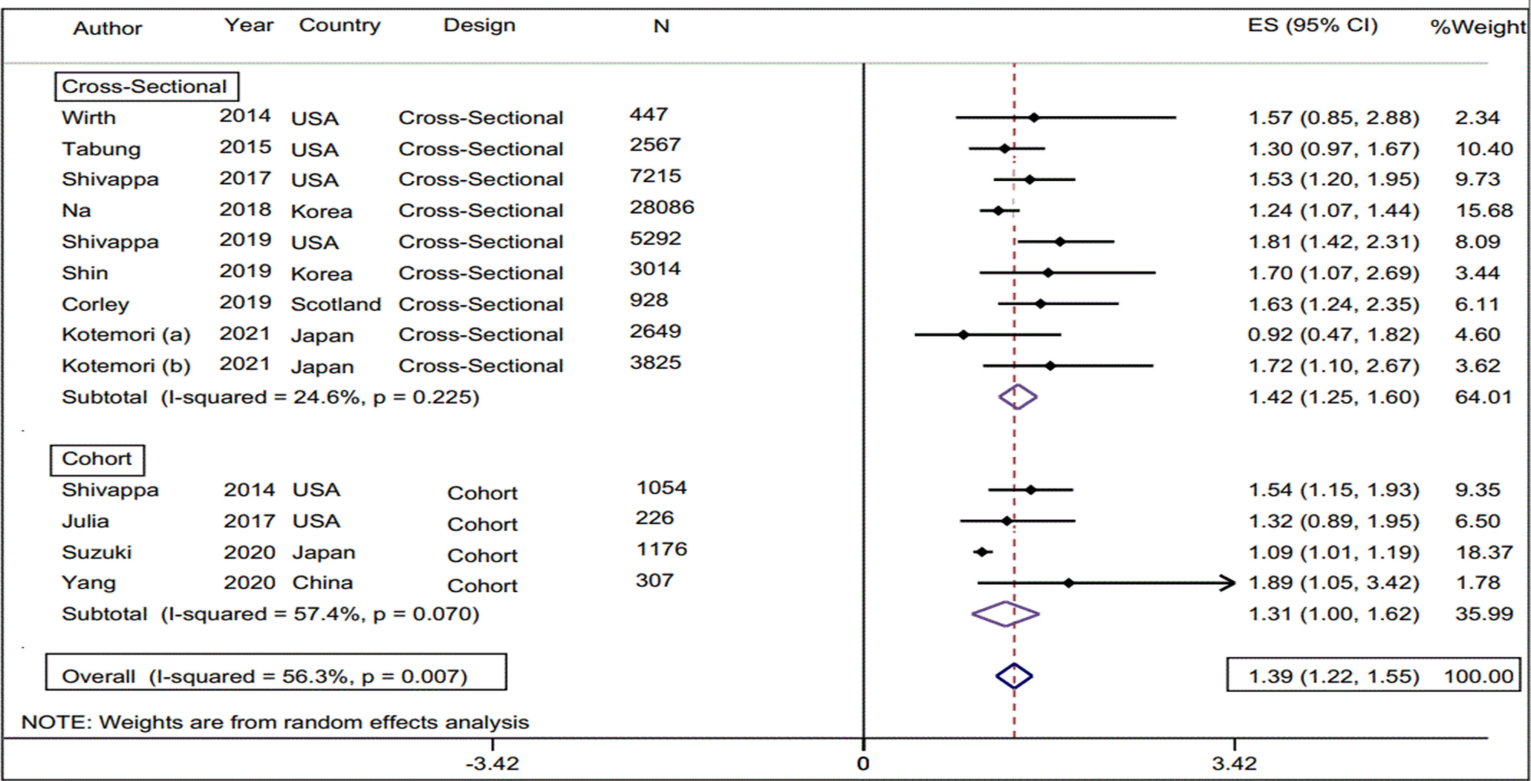
Forest plot of the calculated pooled odds ratio (95% CI) of E‐CRP in the highest DII category in comparison with the lowest DII category.

**FIGURE 3:**
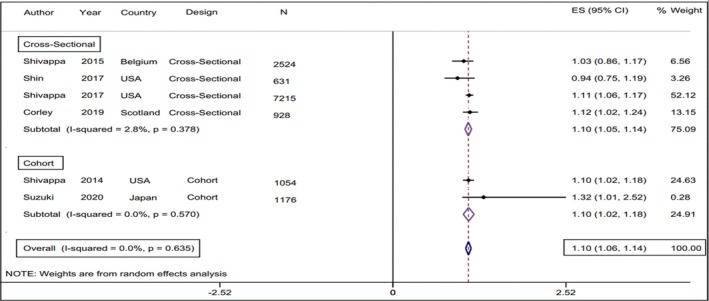
Forest plot of the estimated pooled odds ratio (95% CI) of E‐CRP in relation to DII as a continuous variable.

According to the present study, the calculated pooled odds ratio (OR) of E‐CRP in the highest DII category was 1.39 (95% CI: 1.06, 1.14, test for heterogeneity: *p* = .63, and *I* ^2^ = .0%) in comparison with the lowest DII category (Figure 2). The pooled odds ratio of E‐CRP when DII was considered as a continuous independent variable was estimated at 1.1 (95% CI: 1.06, 1.14, test for heterogeneity: *p* = .63, and *I* ^2^ = .0%) (Figure 3). Subgroup analysis according to study design (cohort, cross‐sectional), gender (male, female), country of origin (USA, Scotland, Belgium, Japan, China, Korea), type of exposure (DII, E‐DII), outcome of interest (CRP, hs‐CRP), DII assessment method (24‐h R, FFQ), CRP assessment method (TBM, NPM), and study adjustments (Energy, BMI) were performed to find potential source of heterogeneity. Subgroup meta‐analyses showed that there is a higher E‐CRP odds ratio for the articles that reported E‐DII instead of DII, the studies that measured CRP instead of hs‐CRP, and the studies that used 24‐h recall instead of FFQ as the instrument of dietary intake data collection. Moreover, this value was 1.74 for men (95% CI 1.14, 2.34), whilst there was no significant between‐category difference in terms of E‐CRP odds ratio in the female population (OR 1.13, 95% CI 0.98, 1.28). The results of the subgroup meta‐analysis are presented in Table 2. According to our findings, the magnitude of the E‐CRP odds ratio in the studies that categorized the DII variable in tertiles was greater than in the studies that examined this variable in quartiles or quintiles (OR = 1.52, 95% CI: 1.06, 1.14, test for heterogeneity: *p* = .78, and *I* ^2^ = .0%) (Table 3).

**TABLE 2:** Meta‐analysis results for odds ratio of CRP positivity including studies reporting DII as a continuous or a categorical independent variable.

Variable	DII‐continuous			DII‐categorical		
	*N* (*n*)^a^	OR (CI 95%)	*I* ^2^% (*p*)	*N* (*n*)^a^	OR (CI 95%)	*I* ^2^% (*p*)
Gender						
Male	1 (650)	1.10 (0.97, 1.23)	–	2 (4475)	1.74 (1.14, 2.34)*	0.0 (0.95)
Female	2 (1157)	0.95 (0.74, 1.16)	0.0 (0.79)	4 (6049)	1.13 (0.98, 1.28)	4.0 (0.38)
Study design						
Cohort	2 (2230)	1.10 (1.05, 1.14)*	0.0 (0.57)	4 (2763)	1.31 (1.00, 1.62)*	57.4 (0.01)
C‐S	4 (11298)	1.10 (1.02, 1.18)*	2.8 (0.38)	9 (54023)	1.42 (1.25, 1.60)*	24.6 (0.22)
Country of origin						
USA	3 (8900)	1.10 (1.05, 1.15)*	7.4 (0.34)	6 (16801)	1.49 (1.32, 1.67)*	0.0 (0.60)
Scotland	1 (928)	1.12 (1.01, 1.23)*	–	1 (928)	1.63 (1.08, 2.18)*	–
Belgium	1 (2524)	1.03 (0.88, 1.18)	–	–	–	–
Japan	1 (1176)	1.32 (0.57, 2.08)	–	3 (7650)	1.13 (0.86, 1.41)	26.1 (0.26)
China	–	–	–	1 (307)	1.89 (0.70, 3.08)	–
Korea	–	–	–	2 (31100)	1.29 (1.01, 1.58)*	14.8 (0.28)
Type of exposure						
DII	5 (12600)	1.10 (1.05, 1.14)*	0.0 (0.52)	9 (44092)	1.31 (1.15, 1.47)*	47.0 (0.06)
E‐DII	1 (928)	1.12 (1.01, 1.23)*	–	4 (12692)	1.56 (1.18, 1.93)*	56.3 (0.01)
Outcome of interest						
CRP	3 (8774)	1.10 (1.04, 1.16)	12.4 (0.32)	5 (14108)	1.58 (1.36, 1.81)*	0.0 (0.73)
hs‐CRP	3 (4754)	1.09 (1.02, 1.16)	0.0 (0.61)	8 (42678)	1.27 (1.10, 1.43)*	45.8 (0.01)
DII assessment method						
24‐h R	3 (8900)	1.10 (1.05, 1.15)*	7.4 (0.34)	7 (45149)	1.46 (1.27, 1.65)*	28.3 (0.21)
FFQ	3 (4628)	1.09 (1.01, 1.18)*	0.0 (0.53)	6 (11592)	1.25 (1.03, 1.47)*	37.6 (0.15)
CRP assessment method						
TBM	–	–	–	4 (14737)	1.46 (1.09, 1.83)*	83.2 (0.0)
NPM	4 (10076)	1.10 (1.06, 1.15)*	0.0 (0.48)	4 (6114)	1.38 (1.11, 1.65)*	67.9 (0.0)
Adjustments						
Energy						
Yes	2 (3700)	1.04 (0.89, 1.19)	0.0 (0.46)	3 (29488)	1.14 (1.03, 1.26)*	56.3 (0.27)
No	3 (8774)	1.10 (1.04, 1.16)*	12.4 (0.32)	10 (27296)	1.52 (1.35, 1.68)*	0.0 (0.62)
BMI						
Yes	4 (11843)	1.11 (1.06, 1.15)*	0.0 (0.72)	10 (54978)	1.36 (1.18, 1.53)*	60 (0.01)
No	1 (631)	0.94 (0.72, 1.16)	–	3 (1808)	1.57 (1.23, 1.92)*	0.0 (0.86)
Overall	6 (13528)	1.10 (1.06, 1.14)*	0.0 (0.63)	13 (56786)	1.39 (1.22, 1.55)*	56.3 (0.01)

Abbreviations: 24‐h R, 24‐hour recall; CRP, C‐reactive protein; C‐S, Cross‐sectional; DII, Dietary Inflammatory Index; E‐DII, Energy‐adjusted Dietary Inflammatory Index; FFQ, Food Frequency Questionnaire; hs‐CRP, High sensitivity C‐reactive protein; NPM, Nephelometry; TBM, Turbidometry.

^a^ *N*, Number of studies; *n*, Pooled sample size.

* Statistically significant at *α* level of 0.05.

**TABLE 3:** Meta‐analysis results for odds ratio of CRP positivity based on subcategories of DII as a categorical variable.

DII subcategories	*N* (*n*)^a^	OR (CI 95%)	*I* ^2^ (%)	*p*‐Value of heterogeneity
Tertile‐3 versus Tertile‐1	4 (2512)	1.52 (1.26, 1.79)*	0.00	0.781
Quartile‐4 versus Quartile‐1	7 (48680)	1.34 (1.12, 1.56)*	66.8	0.006
Quintile‐5 versus Quintile‐1	2 (5581)	1.36 (1.04, 1.68)*	0.00	0.374

Abbreviation: DII, Dietary Inflammatory Index.

^a^ *N*, Number of studies; *n*, Pooled sample size.

* Statistically significant at α level of 0.05.

### 3.2 Publication bias and meta‐regression

**FIGURE 4:**
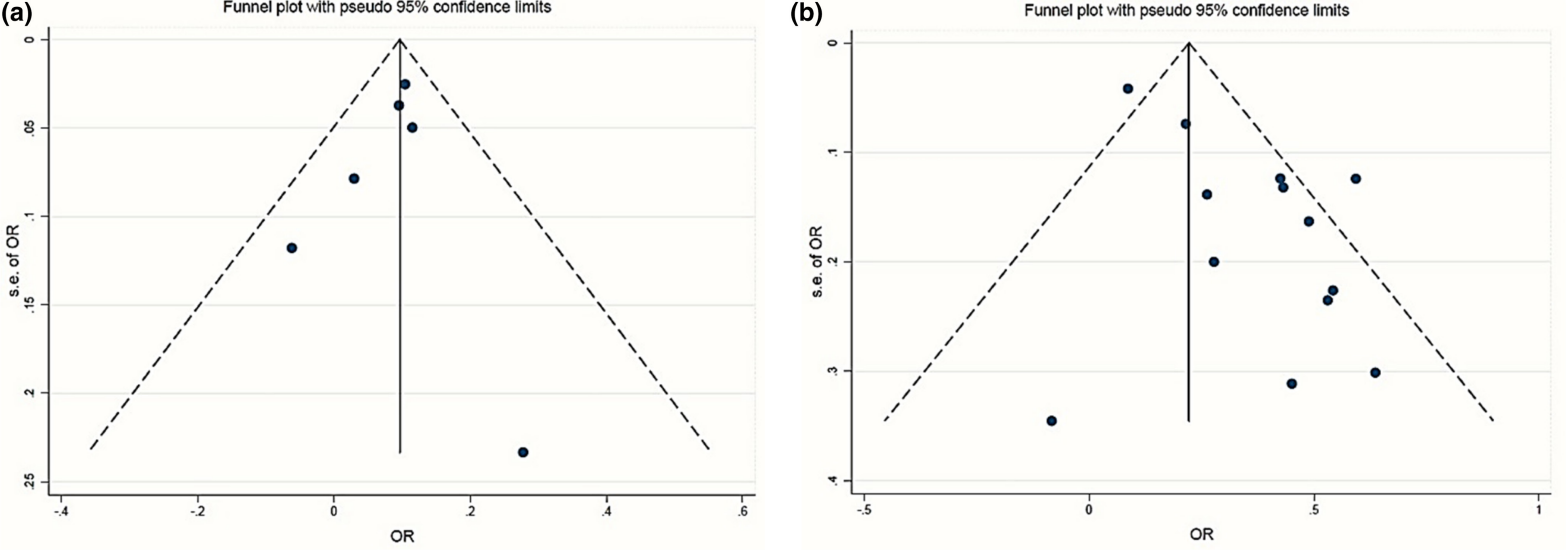
Assessment of publication bias via funnel plot including studies reporting DII as a continuous (a) or a categorical variable (b).

**FIGURE 5:**
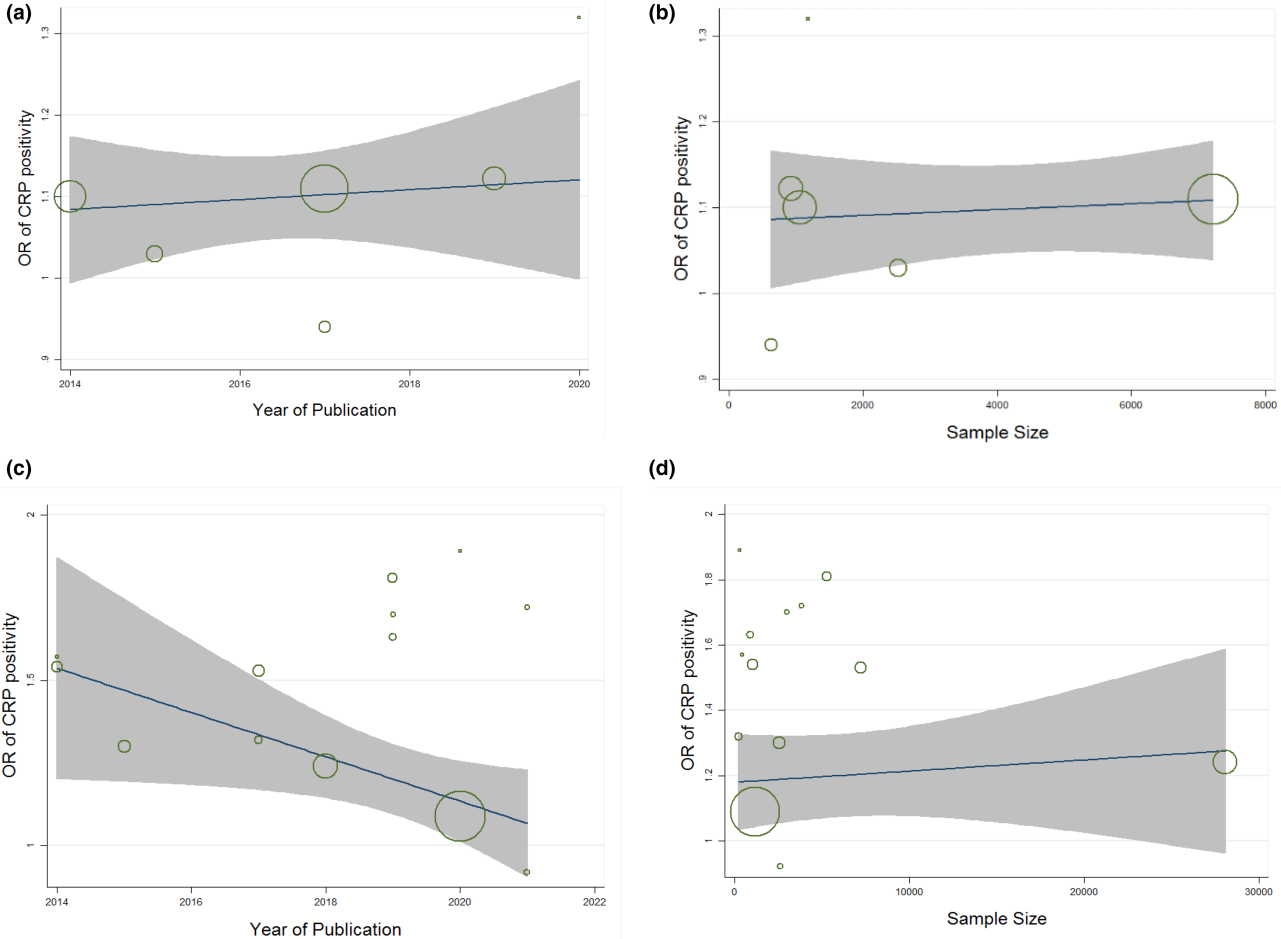
Meta‐regression plots for correlation of CRP positivity odds ratio and sample size or publication year of included studies reporting DII as a continuous (a, b) or a categorical variable (c, d).

Egger's regression test showed that publication bias was not significant for studies that examined the DII variable as a continuous independent variable (*p* = .4). However, we observed a significant publication bias in studies that examined DII as a categorical variable (*p* = .005) but the application of the trim‐and‐fill method did not alter the pooled effect size, indicating that the results were not affected by the publication bias. Funnel plots for visual test of publication bias are also provided in Figure 4. Moreover, the meta‐regression test showed that there was no significant relationship between the E‐CRP odds ratio and the sample size or publication year of included studies (p > 0.05). The corresponding meta‐regression diagrams are shown in Figure 5.

## 4 DISCUSSION

This study showed that higher DII scores were associated with higher odds of having elevated plasma CRP levels in a population of men and women of different nationalities. Thus, our results suggest that adherence to pro‐inflammatory diet that covers low fruits and vegetables, high SFA, and high refined carbohydrates is associated with higher levels of the blood inflammatory biomarker CRP. The findings of this study show that dietary inflammation index plays an important role in changing the inflammatory status of the body, which is congruent with some previous studies, where it has been shown that this index is associated with several health consequences, such as mortality, cardiovascular disease, and kidney disease (Shivappa et al., 2017; Shivappa et al., 2019; M. D. Wirth et al., 2016).

### 4.1 Gender

Our findings showed that the odds ratio of elevated plasma CRP in men in the highest category of DII was approximately 74% higher than the reference category, whilst this difference was statistically insignificant in the female population. In previous studies that have yielded similar results, it has been described as complex to justify this finding because the dietary inflammatory index has been developed based on the results of more than 2000 peer‐reviewed articles, which examined the effects of diet on six inflammatory biomarkers including CRP (Shivappa, Steck, Hurley, Hussey, & Hébert, 2014b; Tabung et al., 2015). However, some studies have considered this difference as a result of the various dietary content of compounds with pro‐inflammatory properties, which considerably differ across men and women. It is evident that the average dietary inflammatory index of women is significantly lower than that of the male population (Kotemori et al., 2021, 2020).

In addition, it has been well established that the presence of chronic diseases, such as diabetes and hypertension, can increase serum CRP levels. In the study of Kotemori et al., which initially did not find a significant relationship between the odds ratio of elevated CRP and DII in the female population, exclusion of the participants consuming chronic antidepressants led to the emergence of a direct and significant correlation between E‐CRP odds ratio and DII score (Kotemori et al., 2021). Furthermore, Suzuki et al. attributed their similar findings to the increased number of female participants with elevated CRP levels in the study population (Suzuki et al., 2020). However, clearly, gender is an important determining factor in the relationship between the dietary inflammatory index and the level of inflammatory biomarkers in serum. Moreover, a dietary inflammatory index for each sex could be created in the future to help provide a more accurate estimate of the diet's impact on the incidence of inflammation in the body.

### 4.2 Study design

Based on our estimates, the participants in the highest category of DII had a greater likelihood of E‐CRP in cross‐sectional studies in comparison with cohort studies. This may be due to the heterogeneity between people in terms of living with different diseases. Serum CRP levels are expected to be higher in people with a variety of diseases than in healthy people. Seemingly, the study population is more heterogenic in terms of disease affliction in cross‐sectional studies than in cohort studies, because in cross‐sectional studies, different demographic characteristics of the population are usually the criteria for inclusion. Meanwhile, in cohort studies, individuals are included if they have not experienced the main outcome, which is usually a disease (Hulley et al., 2013). We also noted that the highest correlation between the odds ratio of E‐CRP and the dietary inflammation index was observed when DII was categorized in tertiles. Although this is highly dependent on the properties of data collected from each population, it seems that categorizing DII data into multiple classes to compare far‐apart categories cannot increase the probability of E‐CRP.

### 4.3 Study origin

According to this meta‐analysis study, the relationship between DII and the odds ratio of E‐CRP was significant in the studies originating in the USA, Scotland, and Korea. Several previous studies have purported that serum CRP level is a function of individuals' nationality. For instance, the mean serum CRP concentration in Japanese men (0.6 mg/L) has been shown to be lower than in the western male population, which is reported to range from 2 to 3 mg/L (Kotemori et al., 2021; Shivappa et al., 2018; Shivappa, Steck, Hurley, Hussey, Ma, et al., 2014a). In addition to the apparent variances in food items consumed in different geographical areas of the world, the food preparation method is another determining factor affecting inflammatory biomarkers, which is a function of the taste, culture, and beliefs of different communities. It is evident that the nutrient content of foods, such as antioxidants and vitamins, can be affected by the temperature and duration of cooking (Loizzo & Tundis, 2022).

Some constant values have been ascribed to the different nutrients of food items based on a primary database of the original version of food composition analyzing software. This database, which is developed in some limited countries with specific cultures, has provided a basis for calculating the dietary inflammation index worldwide. Therefore, software modification according to available food items and conventional cooking methods can be one of the most important solutions to deal with potential errors in calculating the dietary inflammation index in different regions of the world (Víquez et al., 2022). Moreover, a more accurate understanding of this issue can be achieved by conducting more studies in different parts of the world.

### 4.4 Inflammatory biomarker

The present study showed that although the association of DII as a continuous variable with the odds ratio of elevated levels of the two inflammatory biomarkers CRP and hs‐CRP was almost identical, the E‐CRP odds ratio showed a higher association with DII in the studies that examined DII as a categorical variable. So far, several methods have been proposed for the measurement of serum CRP level. Older methods are not able to detect small amounts of CRP due to the high limit of detection, while the hs‐CRP method can detect CRP in values close to 0.5 mg/L (Pearson et al., 2003). It is worth noting that most of the studies that reported hs‐CRP as an inflammatory biomarker were from non‐Western countries, particularly East Asian countries. As mentioned earlier, serum CRP levels in these communities are reported to be lower on average than those in Western countries, which is mainly due to their different dietary and cultural habits. This could affect the magnitude of the relationship between dietary inflammation index and the likelihood of elevated levels of serum inflammatory biomarkers.

The odds ratio of E‐CRP in the highest category of DII was higher when the meta‐analysis was conducted using the studies that measured the inflammatory biomarker by the Nephelometric method than when the studies using the Turbidometric method were included in the meta‐analysis. Although this difference was not considerable (1.46 and 1.38, respectively), it could be a source of heterogeneity in our findings and should be verified in future studies. These methods are used to determine suspended solids in liquids by measuring the light intensity before and after exposure to the sample. In Nephelometry, the intensity of scattered light is measured, while in Turbidometry, the intensity of light passing through the sample is measured (Chianese et al., 2012).

### 4.5 Dietary intake assessment

We found that the magnitude of the E‐CRP odds ratio varied with the instruments of dietary data collection. In other words, this value was considerably higher when including the studies that used 24‐h recall (24HR) vs. FFQ to collect dietary data. This may be due to the different properties of these two data collection instruments. There is evidence that information obtained from 24‐h recall may be more accurate (less biased) estimates than that from FFQ in epidemiological studies (Freedman et al., 2011; Schatzkin et al., 2003). However, the use of the short‐term instrument of 24HR in large cohort studies remains limited due to the costs and logistics of data collection. In addition, a considerable in‐person random error may occur while using 24HR due to the high variability in daily food consumption (Kolar et al., 2005).

In addition, some food items that are important in the calculation of the dietary inflammatory index, such as dark green vegetables, fish, beta‐carotene, or omega‐3 fatty acids, are not often consumed on a daily basis. Therefore, the 24HR short‐term instrument may not provide precise consumption information on these foods. Despite being less accurate than a 24HR, an FFQ has the power to ascertain details about long‐term food consumption. Recently, new technology‐based tools have been developed for dietary data collection in large‐scale studies to overcome the limitations of older instruments including digital image capturing and automated self‐administered 24HRs (Carroll et al., 2012).

### 4.6 Data adjustments

The subgroup meta‐analysis based on BMI adjustment showed that the E‐CRP odds ratio of adjusted subgroup was smaller than that of nonadjusted studies. Body mass index is a strong indicator reflecting the amount of individual energy intake. Among the 45 food parameters used in the calculation of the dietary pro‐inflammatory index, only nine have pro‐inflammatory properties, including energy, carbohydrates, protein, vitamin B12, iron, and some types of fat (total fat, saturated fat, trans fat, and cholesterol) (Shivappa, Steck, Hurley, Hussey, & Hébert, 2014b). Since the increase in BMI occurs due to the preponderance of energy intake over energy expenditure, it is highly probable that people with an increased BMI have an advantage in terms of receiving the aforementioned pro‐inflammatory items that are the main sources of energy in the body (Mohammadi et al., 2021). On the other hand, however, previous studies have shown that serum levels of inflammatory biomarkers, including CRP, may be associated with body mass index (Park et al., 2005). In other words, the simultaneous and independent relationship of BMI to dietary intake and serum inflammatory indices means that this anthropometric index has the necessary characteristics to confound the relationship between DII and serum inflammatory biomarkers (Kamangar, 2012). Therefore, adjustment of BMI, as an important confounding factor, likely better represents reality.

In the present study, we also examined the impact of energy adjustment by conducting two separate models of subgroup meta‐analysis. In the first model, we dichotomized classes based on the type of reported DII variable, namely, E‐DII and DII. For the second model, the studies were classified into adjusted and unadjusted subgroups according to the explanations presented in the article about different ways of energy adjustment. Interestingly, our findings were contradictory in the above two subgroup meta‐analysis, i.e., the odds ratio of E‐CRP in the highest DII category compared to the reference category increased due to energy adjustment in the first model and decreased in the second model. This suggests that, not only the energy intake but also how it is adjusted, can be a source of heterogeneity in epidemiological studies. So far, several methods have been suggested for energy adjustments, one of the most important of which is data analysis based on a constant amount of energy intake (Willett et al., 1997).

### 4.7 Strengths and limitations

As far as we know, this is the first meta‐analysis study to examine the relationship between the E‐ CRP odds ratio and the dietary inflammation index. However, we encountered some limitations. Although all of the studies included in the present meta‐analysis had reported the E‐CRP odds ratio based on the dietary inflammatory index, it is important to note that not all studies included in this meta‐analysis utilized all components of the dietary inflammation index (DII). There were variations in the number of food parameters used to calculate the DII across the included studies, which may have introduced heterogeneity in the results. Moreover, the studies included in the present meta‐analysis had been conducted in a limited number of countries in North America, Europe, and East Asia. Therefore, generalizing the results of this study to the global community is unfeasible, because the eating habits of people from different societies are highly variable under the influence of their cultural and religious beliefs. Additionally, it is important to note that the study protocol was not registered in a trial registry such as PROSPERO, which could have enhanced transparency and reduced potential bias. However, we took measures to ensure rigorous methodology and adherence to established guidelines, such as the MOOSE Guidelines for Meta‐Analyses and Systematic Reviews of Observational Studies.

Despite the aforementioned limitations, there are numerous strengths worth noting. We performed subgroup meta‐analyses based on possible sources of heterogeneity, including the methods of dietary and laboratory data assessment, studies' country of origin, and adjustment of important confounding variables. Also, we performed meta‐regression to examine the possible association between the findings with the sample size and the year of publication of the included studies. However, we had to exclude some studies from the subgroup meta‐analyses due to insufficient information.

## 5 CONCLUSION

We found in the present study that the odds ratio of E‐CRP was higher in the population with higher dietary inflammation index. Men in the highest category of DII were more likely to experience E‐CRP, whilst this value was insignificant in the female population. Also, the odds ratio of E‐CRP, associated with DII, was under the influence of factors such as the nationality of the study population, methods of dietary and laboratory data assessment, study design, and adjustment of BMI and energy. Given that the studies included in the present meta‐analysis were originally from specific regions of the world, it is suggested that well‐designed studies be conducted in the future to provide a more comprehensive insight into the issue of the correlation between diet and inflammatory serum biomarkers.

## AUTHOR CONTRIBUTIONS

**Somaye Saboori:** Conceptualization (lead); project administration (lead); writing – review and editing (equal). **Salman Mohammadi:** Formal analysis (lead); writing – original draft (equal). **Mahboobe Hosseinikia:** Data curation (equal); investigation (equal); writing – review and editing (equal). **Ali Ghaffarian‐Bahraman:** Investigation (equal); writing – original draft (equal). **Cain C. T. Clark:** Validation (equal); writing – review and editing (equal). **Ian Davies:** Validation (equal); writing – review and editing (equal). **Esmaeil Yousefi Rad:** Data curation (equal); investigation (equal); writing – review and editing (equal).

## FUNDING INFORMATION

The current study was funded by Lorestan University of Medical Sciences, Khorramabad, Iran. The funder has played no role in the design of the study or in the collection, analysis, or interpretation of the data.

## CONFLICT OF INTEREST STATEMENT

The authors declare that they have no conflict of interest.

## Supporting information


Table S1

Table S2

## Data Availability

The authors confirm that the data supporting the findings of this study are available within the article.

## References

[fsn33553-bib-0001] Brooke, B. S. , Schwartz, T. A. , & Pawlik, T. M. (2021). MOOSE reporting guidelines for meta‐analyses of observational studies. JAMA Surgery, 156(8), 77–78. 10.1001/jamasurg.2021.0522 33825847

[fsn33553-bib-0002] Calder, P. C. , Ahluwalia, N. , Albers, R. , Bosco, N. , Bourdet‐Sicard, R. , Haller, D. , Holgate, S. T. , Jönsson, L. S. , Latulippe, M. E. , Marcos, A. , Moreines, J. , M'Rini, C. , Müller, M. , Pawelec, G. , van Neerven, R. J. , Watzl, B. , & Zhao, J. (2013). A consideration of biomarkers to be used for evaluation of inflammation in human nutritional studies. British Journal of Nutrition, 109(S1), S1–S34. 10.1017/S0007114512005119 23343744

[fsn33553-bib-0004] Carroll, R. J. , Midthune, D. , Subar, A. F. , Shumakovich, M. , Freedman, L. S. , Thompson, F. E. , & Kipnis, V. (2012). Taking advantage of the strengths of 2 different dietary assessment instruments to improve intake estimates for nutritional epidemiology. American Journal of Epidemiology, 175(4), 340–347. 22273536 10.1093/aje/kwr317 PMC3271815

[fsn33553-bib-0005] Chianese, A. , Bravi, M. , & Fazio, E. (Eds.). (2012). Turbidimetry and nephelometry. In Industrial crystallization process monitoring and control (pp. 51–57). wiley VCH Verlag GmbH & CO. KGaA.

[fsn33553-bib-0006] Corley, J. , Shivappa, N. , Hébert, J. R. , Starr, J. , & Deary, I. (2019). Associations between dietary inflammatory index scores and inflammatory biomarkers among older adults in the Lothian birth cohort 1936 study. The Journal of Nutrition, Health & Aging, 23(7), 628–636. 10.1007/s12603-019-1221-y PMC6675764 31367727

[fsn33553-bib-0007] Esposito, K. , Marfella, R. , Ciotola, M. , Di Palo, C. , Giugliano, F. , Giugliano, G. , D'Armiento, M. , & Giugliano, D. (2004). Effect of a Mediterranean‐style diet on endothelial dysfunction and markers of vascular inflammation in the metabolic syndrome: A randomized trial. JAMA, 292(12), 1440–1446. 15383514 10.1001/jama.292.12.1440

[fsn33553-bib-0008] Freedman, L. S. , Schatzkin, A. , Midthune, D. , & Kipnis, V. (2011). Dealing with dietary measurement error in nutritional cohort studies. Journal of the National Cancer Institute, 103(14), 1086–1092. 21653922 10.1093/jnci/djr189 PMC3143422

[fsn33553-bib-0009] Guo, Y.‐Z. , Pan, L. , Du, C.‐J. , Ren, D.‐Q. , & Xie, X.‐M. (2013). Association between C‐reactive protein and risk of cancer: A meta‐analysis of prospective cohort studies. Asian Pacific Journal of Cancer Prevention, 14(1), 243–248. 23534731 10.7314/apjcp.2013.14.1.243

[fsn33553-bib-0010] Haghani, F. , Arabnezhad, M.‐R. , Mohammadi, S. , & Ghaffarian‐Bahraman, A. (2022). Aloe vera and Streptozotocin‐induced diabetes mellitus. Revista Brasileira de Farmacognosia, 32, 1–14. 10.1007/s43450-022-00231-3 PMC8908758 35287334

[fsn33553-bib-0011] Hulley, S. B. , Cummings, S. R. , Newman, T. B. , Browner, W. , & Grady, D. (2013). Designing cross‐sectional and cohort studies. Designing Clinical Research, 4, 85–96.

[fsn33553-bib-0012] Julia, C. , Assmann, K. E. , Shivappa, N. , Hebert, J. R. , Wirth, M. D. , Hercberg, S. , Touvier, M. , & Kesse‐Guyot, E. (2017). Long‐term associations between inflammatory dietary scores in relation to long‐term C‐reactive protein status measured 12 years later: Findings from the supplementation en Vitamines et Mineraux Antioxydants (SU. VI. MAX) cohort. British Journal of Nutrition, 117(2), 306–314. 28166841 10.1017/S0007114517000034

[fsn33553-bib-0013] Kamangar, F. (2012). Confounding variables in epidemiologic studies: Basics and beyond. Archives of Iranian Medicine, 15(8), 508–516. 22827790

[fsn33553-bib-0014] Kiecolt‐Glaser, J. K. , Derry, H. M. , & Fagundes, C. P. (2015). Inflammation: Depression fans the flames and feasts on the heat. American Journal of Psychiatry, 172(11), 1075–1091. 26357876 10.1176/appi.ajp.2015.15020152 PMC6511978

[fsn33553-bib-0015] Kolar, A. S. , Patterson, R. E. , White, E. , Neuhouser, M. L. , Frank, L. L. , Standley, J. , … Kristal, A. R. (2005). A practical method for collecting 3‐day food records in a large cohort. Epidemiology, 16, 579–583. 15951680 10.1097/01.ede.0000165363.27323.ac

[fsn33553-bib-0016] Kotemori, A. , Sawada, N. , Iwasaki, M. , Yamaji, T. , Shivappa, N. , Hebert, J. R. , Ishihara, J. , Inoue, M. , Tsugane, S. , & JPHC FFQ Validation Study Group . (2020). Validating the dietary inflammatory index using inflammatory biomarkers in a Japanese population: A cross‐sectional study of the JPHC‐FFQ validation study. Nutrition, 69, 110569. 31574409 10.1016/j.nut.2019.110569

[fsn33553-bib-0017] Kotemori, A. , Sawada, N. , Iwasaki, M. , Yamaji, T. , Shivappa, N. , Hebert, J. R. , Potter, J. D. , & Tsugane, S. (2021). Dietary inflammatory index is associated with inflammation in Japanese men. Frontiers in Nutrition, 8, 604296. 33898494 10.3389/fnut.2021.604296 PMC8062774

[fsn33553-bib-0018] Ley, S. H. , Sun, Q. , Willett, W. C. , Eliassen, A. H. , Wu, K. , Pan, A. , Grodstein, F. , & Hu, F. B. (2014). Associations between red meat intake and biomarkers of inflammation and glucose metabolism in women. The American Journal of Clinical Nutrition, 99(2), 352–360. 24284436 10.3945/ajcn.113.075663 PMC3893727

[fsn33553-bib-0019] Loizzo, M. R. , & Tundis, R. (2022). Impact of processing on antioxidant rich foods. In In (Vol. 11, p. 797). MDPI. 10.3390/antiox11050797 PMC9137713 35624661

[fsn33553-bib-0020] Lopez‐Garcia, E. , Schulze, M. B. , Fung, T. T. , Meigs, J. B. , Rifai, N. , Manson, J. E. , & Hu, F. B. (2004). Major dietary patterns are related to plasma concentrations of markers of inflammation and endothelial dysfunction. The American Journal of Clinical Nutrition, 80(4), 1029–1035. 15447916 10.1093/ajcn/80.4.1029

[fsn33553-bib-0021] Medzhitov, R. (2008). Origin and physiological roles of inflammation. Nature, 454(7203), 428–435. 18650913 10.1038/nature07201

[fsn33553-bib-0022] Mohammadi, S. , Rastmanesh, R. , Jahangir, F. , Amiri, Z. , Djafarian, K. , Mohsenpour, M. A. , Hassanipour, S. , & Ghaffarian‐Bahraman, A. (2021). Melatonin supplementation and anthropometric indices: A randomized double‐blind controlled clinical trial. BioMed Research International, 2021, 1–9. 34423033 10.1155/2021/3502325 PMC8373505

[fsn33553-bib-0023] Na, W. , Kim, M. , & Sohn, C. (2018). Dietary inflammatory index and its relationship with high‐sensitivity C‐reactive protein in Korean: Data from the health examinee cohort. Journal of Clinical Biochemistry and Nutrition, 62(1), 83–88. 29371758 10.3164/jcbn.17-22 PMC5773829

[fsn33553-bib-0024] Park, H. S. , Park, J. Y. , & Yu, R. (2005). Relationship of obesity and visceral adiposity with serum concentrations of CRP, TNF‐α and IL‐6. Diabetes Research and Clinical Practice, 69(1), 29–35. 15955385 10.1016/j.diabres.2004.11.007

[fsn33553-bib-0025] Pearson, T. A. , Mensah, G. A. , Alexander, R. W. , Anderson, J. L. , Cannon, R. O., 3rd , Criqui, M. , Fadl, Y. Y. , Fortmann, S. P. , Hong, Y. , Myers, G. L. , Rifai, N. , Smith, S. C., Jr. , Taubert, K. , Tracy, R. P. , Vinicor, F. , & Centers for Disease Control and Prevention; American Heart Association . (2003). Markers of inflammation and cardiovascular disease: Application to clinical and public health practice: A statement for healthcare professionals from the Centers for Disease Control and Prevention and the American Heart Association. Circulation, 107(3), 499–511. 10.1161/01.cir.0000052939.59093.45 12551878

[fsn33553-bib-0026] Peterson, J. , Welch, V. , Losos, M. , & Tugwell, P. (2011). The Newcastle‐Ottawa scale (NOS) for assessing the quality of nonrandomised studies in meta‐analyses. Ottawa Hospital Research Institute.

[fsn33553-bib-0027] Sarwar, N. , Thompson, A. J. , & Di Angelantonio, E. (2009). Markers of inflammation and risk of coronary heart disease. Disease Markers, 26(5–6), 217–225. 19773611 10.3233/DMA-2009-0646 PMC3833412

[fsn33553-bib-0028] Schatzkin, A. , Kipnis, V. , Carroll, R. J. , Midthune, D. , Subar, A. F. , Bingham, S. , Schoeller, D. A. , Troiano, R. P. , & Freedman, L. S. (2003). A comparison of a food frequency questionnaire with a 24‐hour recall for use in an epidemiological cohort study: Results from the biomarker‐based observing protein and energy nutrition (OPEN) study. International Journal of Epidemiology, 32(6), 1054–1062. 14681273 10.1093/ije/dyg264

[fsn33553-bib-0029] Serrano–Martinez, M. , Palacios, M. , Martinez–Losa, E. , Lezaun, R. , Maravi, C. , Prado, M. , Martínez, J. A. , & Martinez–Gonzalez, M. A. (2005). A Mediterranean dietary style influences TNF–alpha and VCAM–1 coronary blood levels in unstable angina patients. European Journal of Nutrition, 44(6), 348–354. 16151968 10.1007/s00394-004-0532-9

[fsn33553-bib-0030] Shin, D. , Hur, J. , Cho, E.‐H. , Chung, H.‐K. , Shivappa, N. , Wirth, M. D. , Hébert, J. R. , & Lee, K. W. (2017). Pre‐pregnancy body mass index is associated with dietary inflammatory index and C‐reactive protein concentrations during pregnancy. Nutrients, 9(4), 351. 28368304 10.3390/nu9040351 PMC5409690

[fsn33553-bib-0031] Shin, D. , Lee, K. W. , Brann, L. , Shivappa, N. , & Hébert, J. R. (2019). Dietary inflammatory index is positively associated with serum high‐sensitivity C‐reactive protein in a Korean adult population. Nutrition, 63, 155–161. 30999247 10.1016/j.nut.2018.11.016

[fsn33553-bib-0032] Shivappa, N. , Bonaccio, M. , Hebert, J. R. , Di Castelnuovo, A. , Costanzo, S. , Ruggiero, E. , Pounis, G. , Donati, M. B. , de Gaetano, G. , Iacoviello, L. , & Moli‐sani study Investigators . (2018). Association of proinflammatory diet with low‐grade inflammation: Results from the Moli‐sani study. Nutrition, 54, 182–188. 29982145 10.1016/j.nut.2018.04.004 PMC6138548

[fsn33553-bib-0033] Shivappa, N. , Hébert, J. R. , Rietzschel, E. R. , De Buyzere, M. L. , Langlois, M. , Debruyne, E. , Marcos, A. , & Huybrechts, I. (2015). Associations between dietary inflammatory index and inflammatory markers in the Asklepios study. British Journal of Nutrition, 113(4), 665–671. 25639781 10.1017/S000711451400395X PMC4355619

[fsn33553-bib-0034] Shivappa, N. , Steck, S. E. , Hurley, T. G. , Hussey, J. R. , & Hébert, J. R. (2014b). Designing and developing a literature‐derived, population‐based dietary inflammatory index. Public Health Nutrition, 17(8), 1689–1696. 23941862 10.1017/S1368980013002115 PMC3925198

[fsn33553-bib-0035] Shivappa, N. , Steck, S. E. , Hurley, T. G. , Hussey, J. R. , Ma, Y. , Ockene, I. S. , Tabung, F. , & Hébert, J. R. (2014a). A population‐based dietary inflammatory index predicts levels of C‐reactive protein in the seasonal variation of blood cholesterol study (SEASONS). Public Health Nutrition, 17(8), 1825–1833. 24107546 10.1017/S1368980013002565 PMC3983179

[fsn33553-bib-0036] Shivappa, N. , Steck, S. E. , Hussey, J. R. , Ma, Y. , & Hebert, J. R. (2017). Inflammatory potential of diet and all‐cause, cardiovascular, and cancer mortality in National Health and nutrition examination survey III study. European Journal of Nutrition, 56(2), 683–692. 26644215 10.1007/s00394-015-1112-x PMC4896851

[fsn33553-bib-0037] Shivappa, N. , Wirth, M. D. , Murphy, E. A. , Hurley, T. G. , & Hébert, J. R. (2019). Association between the dietary inflammatory index (DII) and urinary enterolignans and C‐reactive protein from the National Health and nutrition examination Survey‐2003–2008. European Journal of Nutrition, 58(2), 797–805. 29675557 10.1007/s00394-018-1690-5

[fsn33553-bib-0038] Smidowicz, A. , & Regula, J. (2015). Effect of nutritional status and dietary patterns on human serum C‐reactive protein and interleukin‐6 concentrations. Advances in Nutrition, 6(6), 738–747. 26567198 10.3945/an.115.009415 PMC4642421

[fsn33553-bib-0039] Stang, A. (2010). Critical evaluation of the Newcastle‐Ottawa scale for the assessment of the quality of nonrandomized studies in meta‐analyses. European Journal of Epidemiology, 25(9), 603–605. 20652370 10.1007/s10654-010-9491-z

[fsn33553-bib-0040] Suzuki, K. , Shivappa, N. , Kawado, M. , Yamada, H. , Hashimoto, S. , Wakai, K. , Iso, H. , Okada, E. , Fujii, R. , & Hébert, J. R. (2020). Association between dietary inflammatory index and serum C‐reactive protein concentrations in the Japan collaborative cohort study. Nagoya Journal of Medical Science, 82(2), 237–249. 32581404 10.18999/nagjms.82.2.237 PMC7276400

[fsn33553-bib-0041] Tabung, F. K. , Steck, S. E. , Zhang, J. , Ma, Y. , Liese, A. D. , Agalliu, I. , Hingle, M. , Hou, L. , Hurley, T. G. , Jiao, L. , Martin, L. W. , Millen, A. E. , Park, H. L. , Rosal, M. C. , Shikany, J. M. , Shivappa, N. , Ockene, J. K. , & Hebert, J. R. (2015). Construct validation of the dietary inflammatory index among postmenopausal women. Annals of Epidemiology, 25(6), 398–405. 25900255 10.1016/j.annepidem.2015.03.009 PMC4433562

[fsn33553-bib-0042] Víquez, C. H. , Morales, J. C. , Castro, M. M. , & Herrera, C. C. (2022). Analysis of methodological components and available resources in Costa Rica to generate food composition data. Journal of Food Composition and Analysis, 106, 104294.

[fsn33553-bib-0043] Wannamethee, S. G. , Lowe, G. D. , Rumley, A. , Bruckdorfer, K. R. , & Whincup, P. H. (2006). Associations of vitamin C status, fruit and vegetable intakes, and markers of inflammation and hemostasis. The American Journal of Clinical Nutrition, 83(3), 567–574. 16522902 10.1093/ajcn.83.3.567

[fsn33553-bib-0044] Willett, W. C. , Howe, G. R. , & Kushi, L. H. (1997). Adjustment for total energy intake in epidemiologic studies. The American Journal of Clinical Nutrition, 65(4), 1220S–1228S. 9094926 10.1093/ajcn/65.4.1220S

[fsn33553-bib-0045] Wirth, M. , Burch, J. , Shivappa, N. , Violanti, J. M. , Burchfiel, C. M. , Fekedulegn, D. , Andrew, M. E. , Hartley, T. A. , Miller, D. B. , Mnatsakanova, A. , Charles, L. E. , Steck, S. E. , Hurley, T. G. , Vena, J. E. , & Hébert, J. R. (2014). Association of a dietary inflammatory index with inflammatory indices and the metabolic syndrome among police officers. Journal of Occupational and Environmental Medicine/American College of Occupational and Environmental Medicine, 56(9), 986–989. 10.1097/JOM.0000000000000213 PMC4156884 25046320

[fsn33553-bib-0046] Wirth, M. D. , Shivappa, N. , Hurley, T. G. , & Hébert, J. R. (2016). Association between previously diagnosed circulatory conditions and a dietary inflammatory index. Nutrition Research, 36(3), 227–233. 26923509 10.1016/j.nutres.2015.11.016 PMC4774054

[fsn33553-bib-0047] Yang, Y. , Kan, H. , Yu, X. , Yang, Y. , Li, L. , & Zhao, M. (2020). Relationship between dietary inflammatory index, hs‐CRP level in the second trimester and neonatal birth weight: A cohort study. Journal of Clinical Biochemistry and Nutrition, 66, 19–100. 10.3164/jcbn.19-100 PMC7093294 32231414

