# Supplementary material for: Dietary inflammatory index and elevated serum C‐reactive protein: A systematic review and meta‐analysis
Source: Food Sci Nutr. 2023 Jul 6;11(10):5786–98. doi: 10.1002/fsn3.3553 (PMC10563751; doi:10.1002/fsn3.3553)
Supplement: Supplementary file 1 — Table S1 [file FSN3-11-5786-s002.docx]

| **Concept 1:** | “Dietary inﬂammatory index” OR “inﬂammatory diet” OR “anti-inﬂammatory diet” OR “dietary score” OR DII OR “proinﬂammatory diet” OR “inﬂammatory potential of diet” |
| --- | --- |
| **Concept 2:** | “C-reactive protein” OR “high-sensitivity CRP” OR “hs-CRP” OR CRP |

**Supplementary Table 1**

| PubMed | **(("Dietary inﬂammatory index"[Title/Abstract] OR "inﬂammatory diet"[Title/Abstract] OR "anti-inﬂammatory diet"[Title/Abstract] OR "dietary score"[Title/Abstract] OR DII[Title/Abstract] OR "proinﬂammatory diet"[Title/Abstract] OR "inﬂammatory potential of diet"[Title/Abstract]) OR ("Dietary inﬂammatory index" OR "inﬂammatory diet" OR "anti-inﬂammatory diet" OR "dietary score" OR DII OR "proinﬂammatory diet" OR "inﬂammatory potential of diet"[MeSH Terms])) AND (("C-reactive protein"[Title/Abstract] OR "high-sensitivity CRP"[Title/Abstract] OR "hs-CRP"[Title/Abstract] OR CRP[Title/Abstract]) OR ("C-reactive protein" OR "high-sensitivity CRP" OR "hs-CRP" OR CRP[MeSH Terms]))** | 168 |
| --- | --- | --- |
| WOS | (“Dietary inﬂammatory index”  OR “inﬂammatory diet”  OR “anti-inﬂammatory diet”  OR “dietary score”  OR DII  OR “proinﬂammatory diet”  OR “inﬂammatory potential of diet”) *AND* TOPIC: (“C-reactive protein”  OR “high-sensitivity CRP”  OR “hs-CRP”  OR CRP) | 281 |
| Scopus | (TITLE-ABS-KEY("Dietary inﬂammatory index" OR "inﬂammatory diet" OR "anti-inﬂammatory diet" OR "dietary score" OR DII OR "proinﬂammatory diet" OR "inﬂammatory potential of diet") AND TITLE-ABS-KEY("C-reactive protein" OR "high-sensitivity CRP" OR "hs-CRP" OR CRP)) | 270 |
| All |  | 719 |
