# Supplementary material for: Dietary inflammatory index and elevated serum C‐reactive protein: A systematic review and meta‐analysis
Source: Food Sci Nutr. 2023 Jul 6;11(10):5786–98. doi: 10.1002/fsn3.3553 (PMC10563751; doi:10.1002/fsn3.3553)
Supplement: Supplementary file 2 — Table S2 [file FSN3-11-5786-s001.docx]

**Supplementary Table 2**: Quality assessment of included studies in the current meta-analysis using the Newcastle Ottawa Scale (NOS).

| **Total Score** | **Exposure** | **Outcome** | | | **Comparability** | **Selection** | | | | **Design** | **Study** |
| --- | --- | --- | --- | --- | --- | --- | --- | --- | --- | --- | --- |
|  | | Adequacy of follow up of cohorts | Was follow-up long enough for outcomes to occur | Assessment of outcome | Comparability of cohorts on the basis of the design or analysis | Demonstration that outcome of interest was not present at start of study | Ascertainment of exposure | Selection of the non-exposed cohort | Representativeness of the exposed cohort |  |  |
| 8 |  | * | * | * | ** | -- | * | * | * | Cohort | Julia et al.2017 |
| 7 |  | * | * | * | ** | -- | * | * | -- | Cohort | Shivappa et al.2014 |
| 6 |  | * | * | * | -- | * | * | * | -- | Cohort | Yang et al.2020 |
| 7 |  | * | * | * | * | * | * | -- | * | Cohort | Millar et al.2022 |
|  | | Statistical test | | Assessment of the outcome | The subjects in different outcome groups are comparable, based on the study design or analysis. Confounding factors are controlled | Ascertainment of the exposure (risk factor) | Non-respondents | Sample size | Representativeness of the sample |  | |
| 9 |  | * | | * * | ** | ** | * | * | -- | Cross-Sectional | Corley et al.2019 |
| 8 |  | * | | - | ** | ** | * | * | * | Cross-Sectional | Na et al.2018 |
| 7 |  | * | | * * | ** | ** | * | -- | -- | Cross-Sectional | Shin et al.2017 |
| 10 |  | * | | * * | ** | ** | * | * | * | Cross-Sectional | Shine et al.2019 |
| 10 |  | * | | * * | ** | ** | * | * | * | Cross-Sectional | Shivappa et al.2017 |
| 10 |  | * | | * * | ** | ** | * | * | * | Cross-Sectional | Shivappa et al.2019 |
| 10 |  | * | | * * | ** | ** | * | * | * | Cross-Sectional | Shivappa et al.2015 |
| 6 |  | * | | - | ** | ** | * | -- | -- | Cross-Sectional | Wirth et al.2014 |
| 9 |  | * | | * * | ** | ** | -- | * | * | Cross-Sectional | Tabung et al.2015 |
| 10 |  | * | | * * | ** | ** | * | * | * | Cross-Sectional | Kotemori et al.2021 |
